# SK3 Channel Overexpression in Mice Causes Hippocampal Shrinkage Associated with Cognitive Impairments

**DOI:** 10.1007/s12035-015-9680-6

**Published:** 2016-01-23

**Authors:** Sabine Martin, Marcio Lazzarini, Christian Dullin, Saju Balakrishnan, Felipe V. Gomes, Milena Ninkovic, Ahmed El Hady, Luis A. Pardo, Walter Stühmer, Elaine Del-Bel

**Affiliations:** 1 Department of Molecular Biology of Neuronal Signals, Max Planck Institute of Experimental Medicine, Hermann-Rein-Strasse 3, 37075 Göttingen, Germany; 2 Center Nanoscale Microscopy and Molecular Physiology of the Brain (CNMPB), Göttingen, Germany; 3 Department of Diagnostic and Interventional Radiology, Georg-August University Medical Center, 37075 Göttingen, Germany; 4 Department of Neuro- and Sensory Physiology, Georg-August University Medical Center, 37073 Göttingen, Germany; 5 Department of Pharmacology, Medical School of Ribeirão Preto, University of São Paulo, 14040-900 Ribeirão Preto, Brazil; 6 Department of Neurosurgery, Georg-August University Medical Center, 37075 Göttingen, Germany; 7 Bernstein Focus for Neurotechnology and Bernstein Center for Computational Neuroscience, Göttingen, Germany; 8 Theoretical Neurophysics, Department of Non-linear Dynamics, Max Planck Institute for Dynamics and Self-Organization, 37077 Göttingen, Germany; 9 The Interdisciplinary Collaborative Research Center 889 “Cellular Mechanisms of Sensory Processing”, Göttingen, Germany; 10 Oncophysiology Group, Max Planck Institute of Experimental Medicine, 37075 Göttingen, Germany; 11 Department of Morphology, Physiology and Pathology, CNPQ Research 1B (Biophysics, Biochemistry, Pharmacology and Neuroscience), University of São Paulo Dental School of Ribeirão Preto, Avenida do Café 3400, 14040-904 Ribeirão Preto, Brazil

**Keywords:** Potassium channel KCa2.3, Learning and memory, Whole-cell patch clamp, Schizophrenia, Alzheimer’s disease

## Abstract

The dysfunction of the small-conductance calcium-activated K^+^ channel SK3 has been described as one of the factors responsible for the progress of psychoneurological diseases, but the molecular basis of this is largely unknown. This report reveals through use of immunohistochemistry and computational tomography that long-term increased expression of the SK3 small-conductance calcium-activated potassium channel (SK3-T/T) in mice induces a notable bilateral reduction of the hippocampal area (more than 50 %). Histological analysis showed that SK3-T/T mice have cellular disarrangements and neuron discontinuities in the hippocampal formation CA1 and CA3 neuronal layer. SK3 overexpression resulted in cognitive loss as determined by the object recognition test. Electrophysiological examination of hippocampal slices revealed that SK3 channel overexpression induced deficiency of long-term potentiation in hippocampal microcircuits. In association with these results, there were changes at the mRNA levels of some genes involved in Alzheimer’s disease and/or linked to schizophrenia, epilepsy, and autism. Taken together, these features suggest that augmenting the function of SK3 ion channel in mice may present a unique opportunity to investigate the neural basis of central nervous system dysfunctions associated with schizophrenia, Alzheimer’s disease, or other neuropsychiatric/neurodegenerative disorders in this model system. As a more detailed understanding of the role of the SK3 channel in brain disorders is limited by the lack of specific SK3 antagonists and agonists, the results observed in this study are of significant interest; they suggest a new approach for the development of neuroprotective strategies in neuropsychiatric/neurodegenerative diseases with SK3 representing a potential drug target.

**Electronic supplementary material:**

The online version of this article (doi:10.1007/s12035-015-9680-6) contains supplementary material, which is available to authorized users.

## Introduction

SK3 (KCa2.3, *KCNN3*) belongs to the family of tetrameric, small-conductance calcium-activated potassium channels. At the cellular level, SK3 contributes significantly to the fine-tuning of the duration and amplitude of the action potential after hyperpolarization, regulation of excitability and firing patterns, neurotransmitter release, and synaptic plasticity [1–3]. SK3 is expressed abundantly in the brain including the hippocampus, the limbic system, and midbrain regions rich in monoaminergic neurons [4].

SK3 controls frequency and precision of intrinsic pacemaker activity in dopaminergic neurons [5]. Apamin, a bee venom component that depolarizes dopaminergic neurons by blocking SK channels, facilitates the acquisition of hippocampal-dependent learning tasks in the Morris water maze test [6]. Doxycycline-induced conditional SK3-deficient mice exhibit alterations in cognition tests [7, 8], and SK3 downregulation by antisense reverses age-related deficits in hippocampus-dependent memory tasks and long-term potentiation (LTP) [9]. Conversely, elevated SK3 expression in hippocampi of aged mice contributes to reduced LTP [9] and SK3 overexpressing mice present with impairments in cognition [10]. Finally, the SK3 gene, *KCNN3*, maps to chromosome 1q21, a region containing a major susceptibility locus for schizophrenia, and the polymorphic polyglutamine repeat within this gene is reportedly associated with this illness [11–14]. SK3 contributes to the cognitive abilities of schizophrenic patients, as longer polyglutamine stretches are associated with smaller current amplitude and better cognitive performance [10]. Also, a rare truncation mutant of SK3 (hSK3Δ), originally identified in a patient with schizophrenia, has been found to alter the activity pattern in dopaminergic neurons and reduce attention and sensory gating in mice [15]. Interestingly, the SK3 channel is considered a potential therapeutic target for reducing inflammation-mediated acute central nervous system damage as well as diseases/disorders involving neuron hyper-excitability [16].

A lack of pharmacological agents specific targeting SK3 has mandated the use of a genetic strategy to study the function of this protein. Deignan et al. [17] described SK3 constitutive null mice (SK3-KO) that show a selective impact of SK3 channels on both action potential frequency and timing in dopaminergic neurons. Recently, the same group [18] created a line of SK3 conditional, overexpressing mice (SK3-T/T) through insertion of a doxycycline-sensitive gene switch that permits experimental regulation of SK3 expression while retaining normal SK3 promoter function. Such SK3-T/T mice exhibit increased extracellular striatal dopamine, enhanced hippocampal serotonin release, and reduced hippocampal brain-derived neurotrophic factor (BDNF) expression [7, 8]. In the SK3-T/T mouse line, the transcription of the SK3 channel is reversibly turned off in the presence of the antibiotic doxycycline. Doxycycline at doses [19, 20] used to switch off gene expression is neuroprotective [21] in vitro and in vivo [22–26],

Our aim was to further investigate how SK3 channel expression affects some functions in the central nervous system. For our study, we utilized the SK3 channel overexpressing mice (SK3-T/T [18]), the SK3 constitutive null mice (SK3-KO [17]) and the corresponding wild-type animals (WT). Overexpression of SK3 channel was achieved by maintaining the SK3-T/T mice without doxycycline for several generations. We performed additional behavioral examination and subsequently, and we carried out a detailed macro- and micro-anatomical study in selected regions of the brain.

The results of the study reported here revealed a bilateral reduction in the hippocampal area of the brain of the SK3-T/T mice and additional neurophysiological deficits. We have also been able to delineate unique molecular changes associated with the SK3-T/T mouse brain neurotransmitter systems.

## Materials and Methods

### Animals

The local Animal Ethics Committee approved all experiments (No. AZ 33.9-42502-04-10/0314) and performed according to German law. The SK3 conditionally overexpressing mice [18] and the SK3 constitutive null (knockout) mice [17] were kindly provided by John Adelman and Chris Bond. The SK3 overexpressing allele is referred to as “T” in the following text and homozygotes are indicated as “T/T.” The SK3 null allele is referred to as “-“in the following text and homozygotes are indicated as “KO.” Adult (8–12 weeks) wild-type (WT), heterozygous, and homozygous littermates of both mouse lines were used for all analyses, with the exception of the whole-cell patch clamp recordings, which were taken from CA1 neurons of WT and T/T mice at 30 to 40 days of age.

T/T mice never received doxycycline and these untreated animals show overexpression of the SK3 channel protein throughout development. Only mice, which were referred to as “T/T + DOX”, received doxycycline via food pellets at a dose of 200 mg/kg doxycycline (SSNIFF GmbH, Soest, Germany (www.ssniff.de)); mouse parents were fed with doxycycline 2 weeks before breeding and during the time they were housed together. The mother received doxycycline food until the pups were weaned. Thereafter, the pups received doxycycline until sacrifice.

### Behavioral Study

Male WT, SK3-T/T, and SK3-KO littermates (8–12 animals/genotype) were exposed at the age of 2 months to the novel object recognition (NOR) behavioral test.

The NOR test was carried out in a circular acrylic glass arena (40-cm diameter and 40-cm height). One day before the experiment, each animal was subjected to a 15-min habituation session in the presence of two identical objects. On the experimental day, animals were subjected to two trials spaced by a 1-h interval. During the first trial (acquisition trial, T1), the animals were placed in the arena containing two identical objects for 10 min. For the second trial (test trial, T2), animals were placed back in the arena for 5 min where one of the objects originally presented in the T1 had been replaced by an unknown object (novel object). Behavior was recorded on video for blind scoring of object exploration. Object exploration was defined by: animal licking, sniffing, or touching the object with the forepaws while sniffing. The familiar and novel objects were about 15-cm high, too heavy to be displaced by the animals, but different in shape, color, and texture. Recognition memory was assessed using the discrimination index (discrimination index = (novel − familiar/novel + familiar)), corresponding to the difference between the time exploring the novel and the familiar object, corrected for total time exploring both objects [27].

### Electrophysiological Study

#### Acute Brain Slice Preparation

The animals were anesthetized with isoflurane and brains were quickly removed and transferred into ice-cold buffer containing the following (mM): choline chloride (110), NaHCO_3_ (25), d-glucose (25), Sodium ascorbate (11.6), sodium pyruvate (3.1), KCl (2.5), NaH_2_PO_4_ (1.25), MgSO_4_ (7), and CaCl_2_ (0.5). Coronal slices (350 μm thickness) of whole brain containing the hippocampus were prepared using Leica VT 1200s Vibroslicer and were immersed in artificial cerebrospinal fluid (ACSF) containing (mM): NaCl (126), KCl (3), NaH_2_PO_4_ (1.2), NaHCO_3_ (25), glucose (15), MgCl_2_ (1.1), and CaCl_2_ (2) and continuously bubbled with carbogen (95 % O_2_, 5 % CO_2_). The slices were allowed to recover for 1 h before recording. Afterwards, they were transferred to a submerged chamber and continuously superfused (flow rate of 2–3 ml/min) with ACSF at room temperature.

#### Electrophysiology

Whole-cell patch clamp recording (holding potential, *V*_h_ = −70 mV) were made from CA1 neurons of WT (*n* = 7) and SK3-T/T (*n* = 11) mice of 30 to 40 days of age, under an upright microscope (BX51, Olympus Optical, Tokyo, Japan) equipped with a 40× water-immersion objective and infrared differential interference contrast (IR DIC) illumination. All experiments were performed in the presence of picrotoxin (50 μM) to inhibit GABA_A_ receptors. Baseline stimulation (10 min) was performed at 0.033 Hz with pulses of 0.1 ms width and intensity ranging from 20 to 50 μA. LTP was evoked by a “pairing protocol” consisting of a single 100 Hz tetanus, accompanied by a switch of the *V*_h_ to −10 mV for 1 s, after which the EPSCs were recorded for further 60 min. For patch clamping, borosilicate pipettes of 3–4 MΩ resistance were filled with a solution containing (mM): K-gluconate (110), KCl (5), HEPES (50), EGTA (0.005), MgSO_4_ (4), ATP (4), GTP (0.2), phosphocreatine (9); pH 7.4, 290–300 mOsm/l. Whole-cell currents (EPSCs) were recorded using an EPC 10 amplifier (HEKA Elektronik, Lambrecht, Germany). EPSCs were evoked by stimulating the Schaffer collateral inputs to CA1 neurons using a Teflon-coated platinum electrode placed in the stratum radiatum at a lateral distance of 70–100 μm. Currents were low-pass filtered at 2.5 kHz and sampled at 10 kHz. Series resistance ranged from 10 to18 MΩ and was not compensated. Neurons showing more than 10 % fluctuation in series resistance during LTP measurements were discarded from the analysis.

### Morphological Study

#### MicroCT Imaging System

Brains (7 animals/genotype) were prepared using an adapted phosphotungstic acid (PTA) staining protocol originally described by Metscher et al. [28] and embedded in pairs in 1 % agarose gel to avoid alterations during the imaging session. Samples were imaged using an eXplore Locus SP bench-top microCT (GE Healthcare, Fairfield, USA) operated with the following parameters: 50 kVp tube voltage, 150 μA tube current, and 1800 angular projection within a full rotation. For each single sample, a 3D data set was reconstructed with an isotropic voxel size of 16 μm. On virtual coronal cross-sections at the position of Bregma −1.70 mm, the combined hippocampus ventricle area (HV.Area) as well as the ratio of the hippocampus area to HV.Area (H.Ratio) were measured using the 3D rendering and analysis software Scry (v5, Kuchel and Sautter GbR). Due to the high contrast provided by the PTA staining, the total brain volume was assessed using a threshold-based segmentation to separate the brain tissue from the agarose gel. In order to account for partial volume effects, the arithmetic mean between the average attenuation value of the brain and the agarose gel was chosen as the threshold. To negate any influence of the different sizes of the analyzed mice, the tibia length (TL) was used as a reference and measured using the same microCT. Therefore, the HV.Area was normalized by TL^2 and B.Vol by TL^3. H.Ratio that is already size independent does not require normalization. Finally, to facilitate the presentation of the results, the values are expressed as ratio to the mean value of the respective measurements of the WT control group. Therefore, mean WT is always 1 and the graphs display the ratio to this base value.

### Molecular Study

For immunohistochemistry (4 animals/genotype), quantitative RT-PCR analysis (3–7 animals/genotype), and PCR array (4 animals/genotype), brain tissues were dissected and analyzed.

#### Immunohistochemistry

The animals were deeply anesthetized by intraperitoneal injection of ketamine and xylazine and transcardially perfused with 30 ml of PBS, followed by 30 ml of PBS with 4 % paraformaldehyde (PFA) (Sigma-Aldrich, St. Louis, USA), pH 7.4. Brain tissues were carefully dissected, postfixed overnight with 4 % PFA at 4 °C, and cut in a vibratome (VT1000S; Leica, Wetzlar, Germany; 30-μm sections) according to Lazzarini et al. [21]. For NeuN immunostaining, sections were incubated using mouse monoclonal anti-NeuN (1:1000; Millipore, Billerica, USA). Species-specific secondary biotinylated antibody IgG (1:500; Vector, Burlingname, USA) was used. After incubation with the avidin–biotinylated horseradish peroxidase complex ABC (Vector), the immunocomplex was visualized by the substrate 3,3′-diaminobenzidine tetrahydrochloride (DAB, 1 mg/ml; Sigma-Aldrich). Bright field images were obtained with an Axiovert 200 M microscope (Zeiss, Oberkochen, Germany).

#### Quantitative RT-PCR of *DRD1A* and *DRD2*

The animals were sacrificed by CO_2_ inhalation and subsequently decapitated. Total RNA from brain regions was obtained as described by Martin et al. [29]. The relative abundance of dopamine (DA) receptor type 1 (*DRD1A*) and DA receptor type 2 (*DRD2*) transcripts in frontal cortex, dorsal and ventral striatum, hippocampus, mesencephalon, and amygdala of mouse brain were studied by quantitative RT-PCR. The Ct value of these target genes was normalized to the reference genes hypoxanthine guanine phosphoribosyl transferase 1 (*HPRT1*) and hydroxymethylbilane synthase (*HMBS*). For quantifying mRNA expression by real-time PCR, the following fragments were amplified: nt 134–233 from sequence NM_013556 detected with the m*HPRT1* probe (5′-(Fam)-CAGCGTCGTGATTAGCGATGATGAACCAGG-(Tamra)-3′); nt 476–587 from sequence NM_013551 detected with the m*HMBS* probe (5′-(FAM)-ACTATTGGAGCCATCTGCAAACGGGA-(Tamra)-3′); nt 1576–1675 from sequence NM_010076 detected with the m*DRD1A* probe (5′-(Fam)-CAACAACAACGGGGCTGTGATGTTTTCCA-(Tamra)-3′); nt 607–706 from sequence NM_010077 detected with the m*DRD2* probe (5′-(Fam)-CTCTTTGGACTCAACAACACAGACCAGA-(Tamra)-3′).

Conditions for PCR were 2 min at 50 °C, 10 min at 95 °C, 15 s at 95 °C, 15 s at 56 °C, and 1 min at 60 °C (50 cycles).

#### PCR Array of Dopamine and Serotonin (5HT) and Glutamate and Gamma-Aminobutyric Acid Pathways

qPCR was performed using ready-to-use mouse DA/serotonin (5HT) and gamma-aminobutyric acid (GABA)/glutamate (GLU) pathway RT^2^ Profiler PCR array (Qiagen/SABiosciences, Hilden, Germany; Cat. No. PAMM-158Z and PAMM-152Z) containing primers for 84 target and 5 housekeeping genes and controls for RT and PCR reactions. cDNA isolated from hippocampus was applied to these commercially available plates.

### Statistical Analysis

#### Behavioral Data

The data for the NOR test were normally distributed and permitted two-way multivariate analysis of variance for parametric test. Therefore, NOR was analyzed by two-way multivariate analysis of variance (MANOVA) with the genotype as the independent factor. Bonferroni post hoc test was used as indicated to specify differences revealed by significant MANOVAs. *P* < 0.05 was considered significant. Statistical behavior analysis was performed using the SPSS v.8.0 software.

#### Electrophysiological Data

Data points were normalized to the mean EPSC amplitude during baseline sampling to create time kinetics graphs. Results are presented as mean ± SEM. Representative EPSCs are averages of five consecutive traces. LTP was calculated as the mean of normalized EPSC amplitudes during the last 5 min of recording of individual neurons. Statistical comparison was achieved using the Student’s *t* test.

#### MicroCT

The difference of the combined hippocampus ventricle area (HV.Area), the ratio of the hippocampus area to HV.Area (H.Ratio) and the total brain volume were analyzed using a one-way ANOVA implemented in the statistic software PAST [30]. *P* < 0.05 was considered significant.

#### Immunohistochemistry

Analysis was performed using independent *t* test for parametric data comparing WT and T/T animal groups. *P* < 0.05 was considered significant.

#### RT-PCR

The differences in the normalized mRNA content were analyzed by independent *t* test for parametric data comparing the wild-type group with the transgenic mouse group. *P* < 0.05 was considered significant.

#### PCR Array

Data were analyzed using the manufacturer (Qiagen/SABiosciences) web-based software (http://pcrdataanalysis.sabiosciences.com/pcr/arrayanalysis.php).

## Results

### Cognitive/Memory Deficits in SK3-T/T Mice

The overexpression and absence, respectively, of *KCNN3* mRNA or SK3 protein were confirmed in all T/T and KO mice by RT-PCR and Western blot (data not shown).

In experiments to address the cognitive consequences of SK3 overexpression and deficiency, we used the SK3-T/T and SK3-KO mice, respectively. Grube et al. [10] and Jacobsen et al. [7, 8] have shown a role of the KCNN3 gene and the SK3 potassium channel in cognitive function.

SK3-KO and WT mice displayed the expected [31] preference in the novel object recognition test for the novel object in the retention trial (results not shown).

**Fig. 1:**
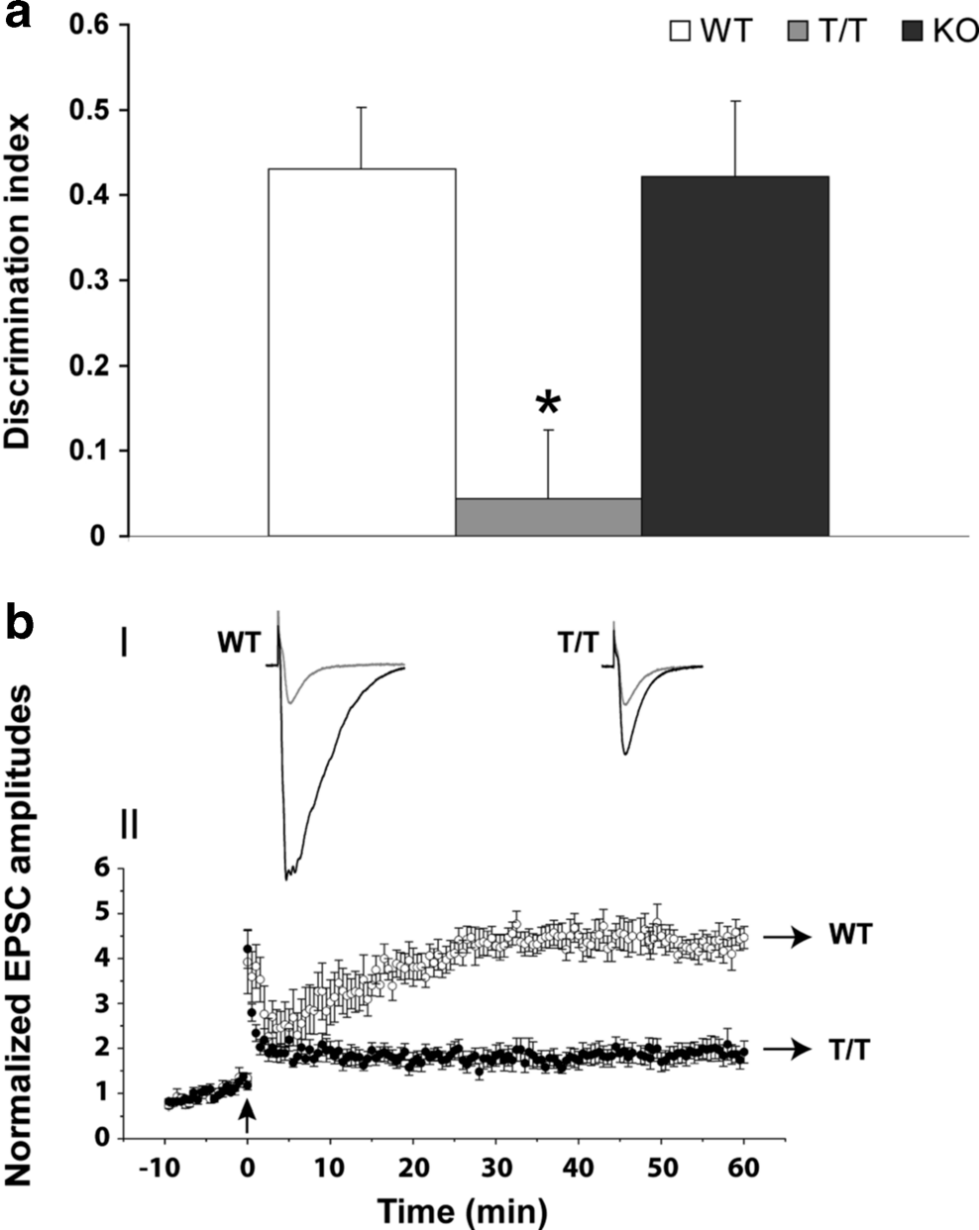
T/T mice show cognitive and memory impairments and reduced LTP. **a** Behavioral NOR test was performed in independent groups of male WT (*n* = 8–12), T/T (*n* = 8–12), and KO (*n* = 8–12) littermates. T/T presents less discrimination index (*P < 0.05) than WT and KO mice. Statistical analysis was performed using two-way MANOVA with Bonferroni test for NOR. **b** LTP of CA1 pyramidal neurons from WT (*n* = 7) and age matched T/T (*n* = 11) mice. (*I*) Representative EPSCs under control conditions (*grey*) and during LTP (*black*) from WT and T/T mice. Traces are averages of five consecutive sweeps. Stimulus artifacts are truncated for clarity. (*II*) Time kinetics of normalized EPSC amplitudes of CA1 neurons from WT and T/T mice. Data points from −10 to 0 min represent baseline amplitudes. Initial stimulations of Schaffer collateral afferents lasted 10 min to obtain baseline EPSC amplitude of ~100 pA. The pairing protocol to induce LTP was applied at the same current intensity as the baseline stimulation. A brief stimulus consisting of 100 Hz tetanus and simultaneous voltage step to 0 mV for 1 s at 0 min (indicated by a *black arrow*) induced a larger LTP (4.37 ± 0.05 times of baseline (*P* < 0.0001, single-sample *t* test) that was stable for at least 60 min (longest period recorded) in WT mice (*open circles*) compared to T/T. T/T mice (*closed circles*) have significant less LTP (1.93 ± 0.06 times of baseline, *P* < 0.0001, single-sample *t* test)

In contrast, SK3-T/T mice failed to show novel object preference (two-way MANOVA, *F*(2,26) = 48.96; Bonferroni test, *P < 0.05; Fig. 1a), confirming a cognitive impairment.

The results presented above support converging evidence that SK3 channels regulate cellular mechanisms of memory encoding [32].

### Reduced Long-Term Potentiation in the CA1 Hippocampus of SK3-T/T Mice

Elevated expression of SK3 channel in hippocampi of aged mice is known to contribute to reduced long-term potentiation [9] and gene-silencing of SK3 resulting in short-term memory problems [7, 8].

To analyze functional deficits in synaptic transmission, we prepared hippocampal slices of WT and T/T mice of 30 to 40 days of age. Whole-cell patch clamp recordings were made from CA1 neurons.

CA1 neurons from WT had a resting membrane potential (Vm) of −72.3 ± 0.4 mV, not significantly different from the T/T CA1 neurons (−72.0 ± 0.3 mV; *n* = 26, *P* = 0.61, two-sample *t* test). WT and T/T CA1 neurons also displayed similar input resistance (68.2 ± 1.5 and 63.0 ± 1.9 MΩ, respectively, *n* = 20, *P* = 0.09, two sample *t* test).

SK3-T/T mice presented with remarkable LTP deficits (Fig. 1bI, bII). A brief stimulus consisting of 100 Hz tetanus and simultaneous voltage step to −10 mV for 1 s at 0 min [Fig. 1bII, indicated by an arrow] induced a large LTP (4.37 ± 0.05 times baseline (*P* < 0.0001, single-sample *t* test) stable for 60 min (longest period recorded) in WT animals (open circles). SK3-T/T animals in contrast presented a LTP two times smaller (closed circles; 1.93 ± 0.06 times of baseline, *P* < 0.0001, single-sample *t* test).

### Morphological Changes in the Brain of SK3-T/T Mice

**Fig. 2:**
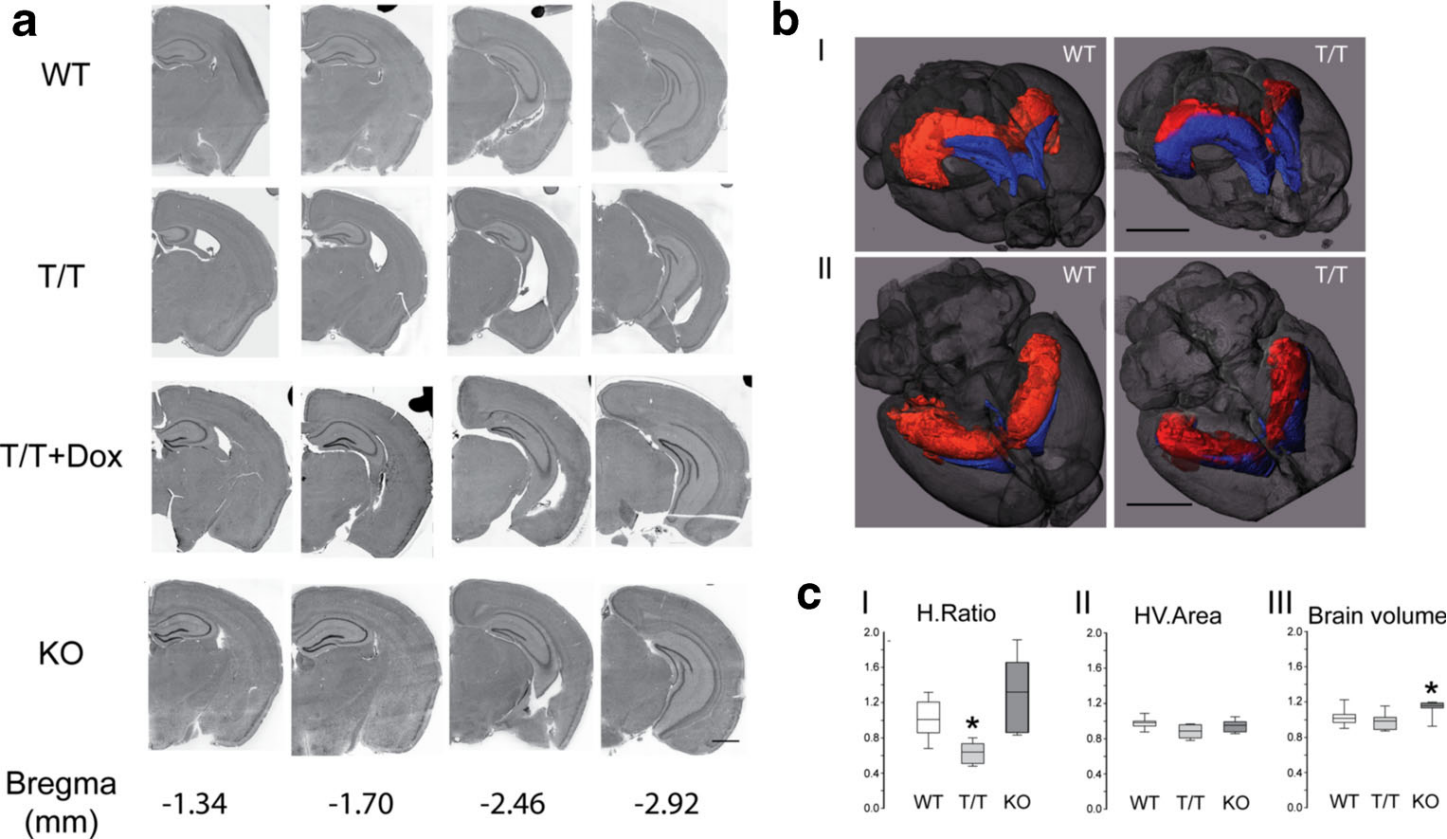
SK3 channel overexpression induces morphological changes in hippocampus. **a** Brain coronal sections (vibratome, 30 μm) of WT (*n* = 5), T/T (*n* = 5), doxycycline treated T/T (T/T + DOX, *n* = 5), and KO (*n* = 5) mice. Four brain levels were chosen (Bregma −1.34 to −2.92 mm) for hematoxylin-eosin staining analysis. Simple observation reveals a marked decrease of the hippocampus size and an enlargement of the lateral ventricles (Bregma −1.34, −1.70, and −2.46 mm) with no obvious changes at Bregma −2.92 mm. WT, T/T + DOX, and KO mice show no hippocampal shrinkage. *Scale bar*, 1 mm. **b**, **c** MicroCT analysis of WT (*n* = 7), T/T (*n* = 7), and KO (*n* = 7; C) mice. **b** Three-dimensional (3D) rendering representation of a stained WT and T/T mouse brain with rostral-dorsal (*I*) and dorsal (*II*) view scanned with the eXplore Locus SP bench-top microCT. Hippocampal region and lateral ventricle were segmented separately using a region-growing algorithm and are displayed in *red* (hippocampus) and *blue* (ventricles), respectively. The brain surface is displayed semi-transparent in *grey*. Whereas the combined structure of ventricle and hippocampus appears to have the same volume in both WT and T/T samples, the ventricles are clearly enlarged and therefore the hippocampus shows shrinkage in the T/T mouse brain. *Scale bars*, 3 mm. **c** On virtual coronal cross sections at the position of XYZ, the ratio of the hippocampus area to the combined hippocampus plus ventricle area (HV.Area; H.Ratio, *I*), the HV.Area (*II*) as well as the brain volume (*III*) were measured using the 3D rendering and analysis software Scry (v5, Kuchel and Sautter GbR). The analyses confirmed the reduction of hippocampal area (H.Ratio) in the T/T group compared to the other groups (*I*). No differences in the HV.Area were found (*II*). The brain volume of KO mice was slightly increased (*III*). The values are expressed as ratio to the mean value of the respective measure of the WT control group (one-way ANOVA, *P < 0.05)

The dissection of brain regions for qRT-PCR analysis revealed that T/T mice show dramatic brain deformity. Careful observation indicated that SK3 overexpressing T/T mice exhibit bilateral hippocampal shrinkage (more than 50 %), mostly in the rostral part of the brain producing an outsized lateral ventricle (Fig. 2a). The hippocampus was replaced by a large cavity contiguous with the ventricular system.

In order to analyze if the observed hippocampal shrinkage was a product of the inserted doxycycline-sensitive gene switch, we fed a group of T/T mice with dietary doxycycline (group T/T + DOX) during embryogenesis to adulthood. This largely abolished channel expression [18] and restored the hippocampus to normal size (Fig. 2a) suggesting that the morphological changes were dependent on SK3 overexpression. Indeed, no morphological changes were detected in SK3 knockout (KO) mice (Fig. 2a).

Microcomputed tomography (microCT) analysis confirmed the reduction of the hippocampal area (H.Ratio; one-way ANOVA, *P < 0.05) observed through the anatomical dissection in the T/T mice compared to the other groups (Fig. 2bI, bII, cI; as shown in the Supplementary Movies 1a and b (Online Resource)). There were no differences in either of the combined hippocampus plus ventricle area (HV.Area) (Fig. 2cII) or the brain volume between T/T and WT mice (Fig. 2cIII). Structural changes did not result from differences in overall body size because the measured tibia length did not show differences between groups. There was a small increase in the brain volume of KO mice when compared with the WT littermates (Fig. 2cIII).

### Cellular Characterization of the Hippocampal Formation of SK3-T/T Mice

**Fig. 3:**
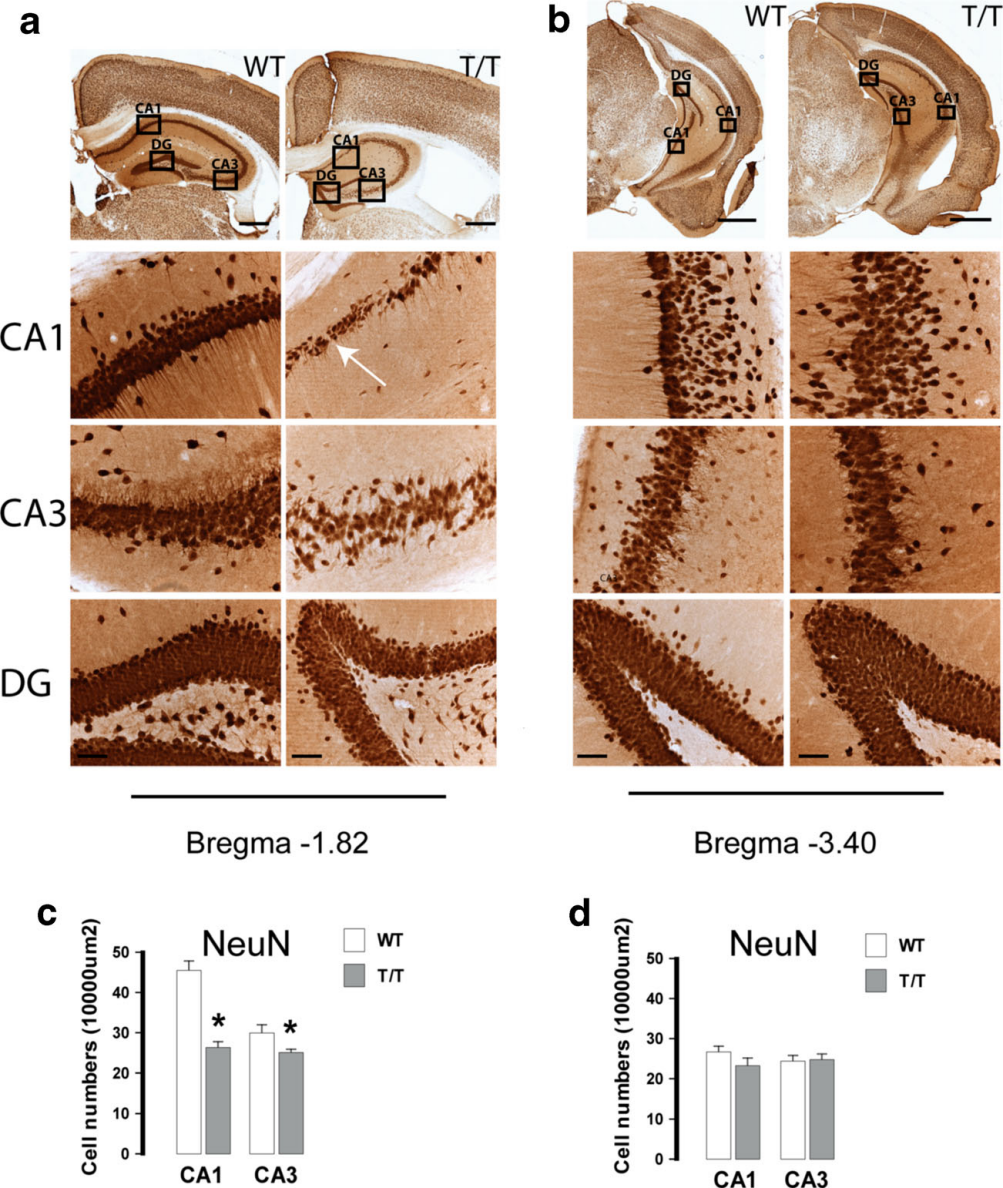
T/T mice show neuronal loss and disorganized neurons in the CA1 and CA3 region. **a**, **b** Micrographs of neuronal nuclei (NeuN)-positive cells in subregions of hippocampus (CA1, CA3, DG) from rostral (**a**) and caudal (**b**) hippocampus of WT (*n* = 4) and T/T (*n* = 4) mice. *Insets* show magnifications of stained granule neurons in dentate gyrus (DG) and pyramidal neurons in Ammon’s horn CA1 and CA3. There are heterotopias (misplaced neurons) and cell discontinuities (disruption in the cornu ammonis [CA] layers) indicated by a *white arrow* (**a**, T/T-CA1). There were dispersed NeuN-positive neurons on all sides of the CA1 cellular layer. The cells of the pyramidal layer in rostral CA1 are less densely packed and the column organization is less obvious. *Scale bars*, 0.5 mm in **a**, 1 mm in **b**; *insets*, 50 μm in **a** and **b. c**, **d** Quantifications of NeuN-positive cells in rostral (**c**) and caudal (**d**) hippocampus are shown as the normalized average and SDs of three independent replicates. T/T mice show reduced amount of NeuN-positive cells in both CA1 and CA3 of rostral hippocampus (Bregma −1.82 mm; independent *t* test, *P < 0.05). Values represent number of neurons per 10,000 μm^2^ (mean ± SEM)

Analysis of hippocampal neuronal cytoarchitecture by NeuN staining revealed well-defined layers in the entorhinal cortex and in the cornu ammonis (CA) of control animals, whereas somas and axon hillocks showed a columnar and parallel organization, respectively (Fig. 3a, b). In comparison, analysis of T/T mice hippocampi revealed a much less strict cellular organization with dispersed neurons on either side of the pyramidal cellular layer. Interruptions in the neuronal layer continuity [white arrow in Fig. 3a] were observed in the CA1. The cells of the pyramidal layer in CA1 were less densely packed and the columns were less obvious (Fig. 3a, b).

Neuronal density was calculated as the number of labeled NeuN-positive neurons divided by the area (10,000 μm^2^) of interest. The number of neurons decreased in both CA1 and CA3 in rostral hippocampi (Bregma −1.82 mm) of T/T mice (Fig. 3c; independent *t* test, *P < 0.05), whereas in the caudal area (Bregma −3.40 mm), no differences were found compared to WT (Fig. 3d).

Out of 60 T/T animals analyzed, two individuals did not show evident hippocampal shrinkage. Interestingly, these animals still exhibited the neuronal loss and disruption in the CA layer (data not shown).

### Molecular Alterations in the Brain of SK3-T/T Mice

Mutations in some ion channels known to regulate dopaminergic neuron physiology have been linked to several central nervous system illnesses [33–37]. Major hypotheses link alterations of dopamine, serotonin (5HT), glutamate (GLU), gamma-aminobutyric acid (GABA), and calcium pathways with known risk-associated genes [38].

**Fig. 4:**
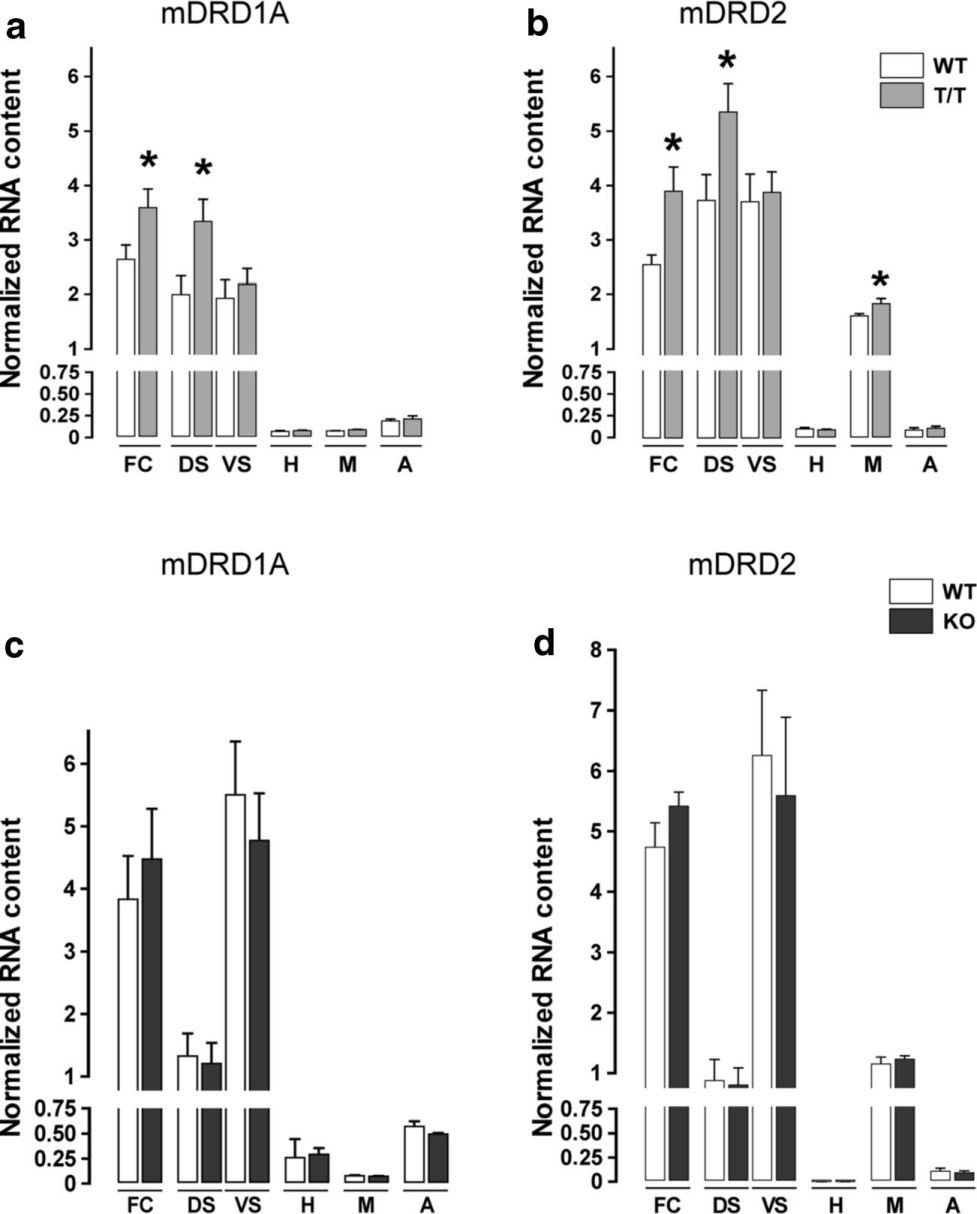
T/T mice exhibit gene expression changes in the dopamine receptors type 1a and 2 in different brain regions. **a**, **b** qRT-PCR analysis of *DRD1A* (**a**) and *DRD2* (**b**) in frontal cortex (*FC*), dorsal (*DS*), and ventral striatum (*VS*), hippocampus (*H*), mesencephalon (*M*), and amygdala (*A*) of WT (*n* = 7) and T/T (*n* = 7) mice. There is an upregulation of *DRD1A* in FC and DS and of *DRD2* in FC, DS, and M of T/T mice in comparison to WT (independent *t* test, *P < 0.05). **c**, **d** qRT-PCR analysis of *DRD1A* (**c**) and *DRD2* (**d**) in frontal cortex (*FC*), dorsal (*DS*), and ventral striatum (*VS*), hippocampus (*H*), mesencephalon (*M*), and amygdala (*A*) of WT (*n* = 7) and KO (*n* = 7) mice. There are no differences in mRNA expression of *DRD1A* and *DRD2* in KO mice in comparison to WT

We performed qRT-PCR focused on dopamine receptors D1 and D2 in different brain regions of T/T, KO, and WT mice. cDNA from the frontal cortex, dorsal, and ventral striatum, hippocampus, mesencephalon, and amygdala was analyzed using specific TaqMan probes to determine *DRD1A* and *DRD2* expression levels. There was an upregulation of DA receptors *DRD1A* and *DRD2* in the frontal cortex and dorsal striatum and of *DRD2* in the mesencephalon in T/T mice (Fig. 4a, b; independent *t* test, *P < 0.05).

In the case of KO mice, we did not find any difference compared to WT (Fig. 4c, d).

### Molecular Alterations in the Hippocampus of SK3-T/T and SK3-KO Mice

We further performed two PCR array analyses in the hippocampus of SK3-T/T mice exploring the DA/5HT and the GABA/GLU pathways.

**Fig. 5:**
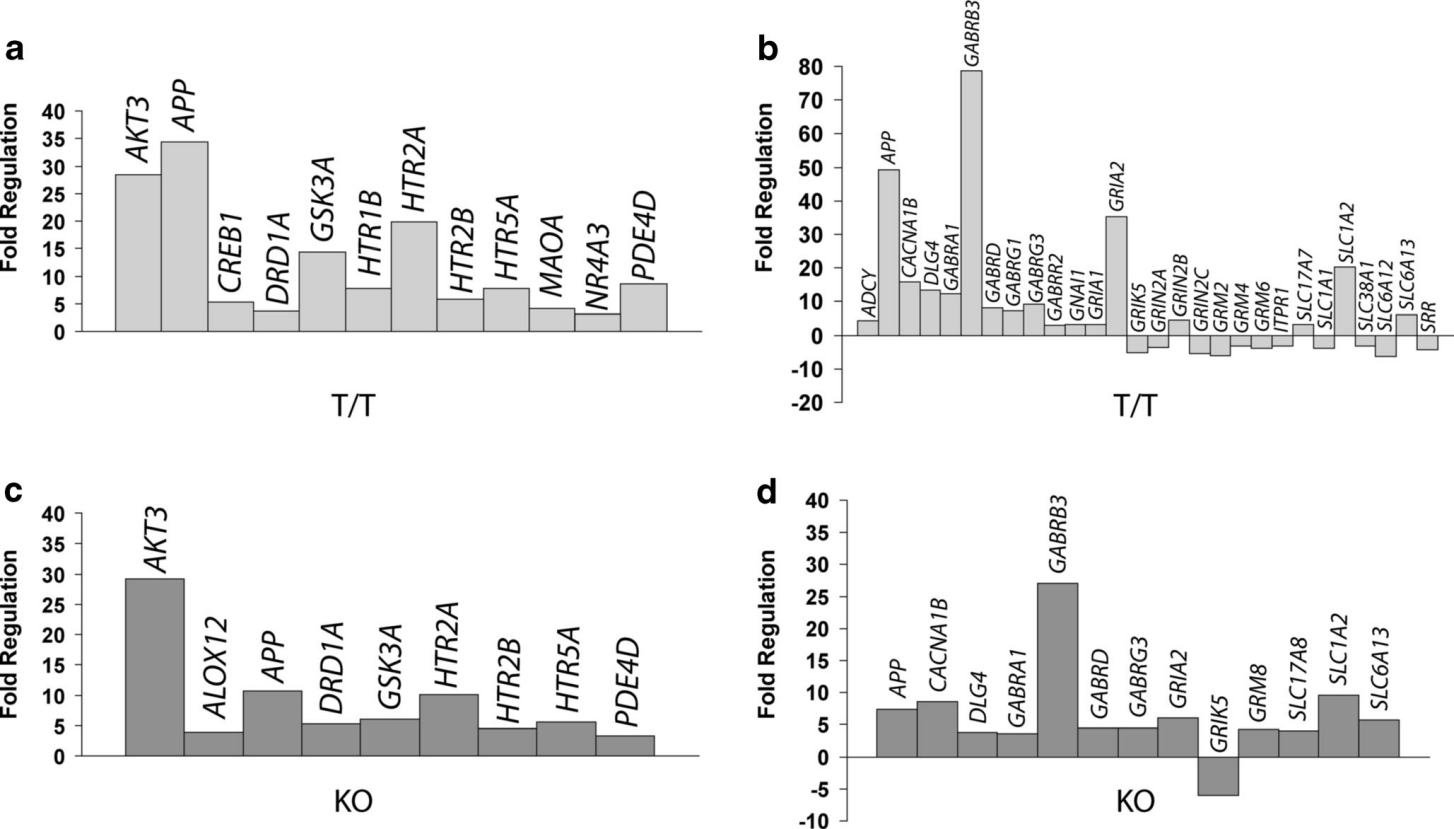
T/T mice exhibit hippocampal gene expression changes in the DA and 5HT and GABA and GLU pathways. **a**, **b** Expression analysis in the hippocampus of T/T (*n* = 4) versus WT (*n* = 4) mice using a PCR array of the DA/5HT (**a**) and of the GABA/GLU (**b**) pathways reveals an upregulation of (**a**) *AKT3*, *GSK3A*, and *HTR2A* and (**b**) *GABRB3*, *GRIA2*, and *SLC1A2* mRNA. There is also an increase in the abundance of *APP* mRNA in T/T mice in both neurotransmitter pathways. **c**, **d** Expression analysis in the hippocampus of KO (*n* = 4) versus WT (*n* = 4) mice using a PCR array of the DA/5HT (**c**) and of the GABA/GLU (**d**) pathways reveals an upregulation of (**c**) *AKT3* and (**d**) *GABRB3* mRNA. Note that in the hippocampus of KO mice, *AKT3* shows a similar upregulation and *GABRB3* is slightly increased in comparison to T/T mice

The results of the DA and 5HT pathways analysis in SK3-T/T indicated a strong upregulation of the thymoma viral proto-oncogene 3 (*AKT3*), the glycogen synthase kinase 3 alpha (*GSK3A*), and the 5HT2A receptor (*HTR2A*) genes (Fig. 5a).

The GABA and GLU pathways analysis in SK3-T/T showed a major increase in mRNA expression of the GABA-A receptor subunit beta 3 (*GABRB3*), the ionotropic glutamate receptor AMPA2 (alpha 2-*GRIA2*), and the solute carrier family 1 (glial high-affinity glutamate transporter) member 2 (*SLC1A2*) (Fig. 5b).

There was a consistent increase in the abundance of mRNA encoding Alzheimer’s disease amyloid precursor protein (*APP*) in T/T mice in both arrays (Fig. 5a, b).

The genes of the DA/5HT or GABA/GLU pathways that we analyzed above also showed highly upregulated expression in the hippocampus of T/T mice (Fig. 5a, b), but showed no prominent expression changes in the hippocampus of KO in comparison to WT mice (Fig. 5c, d). The only exception was the gene *AKT3* that showed a similar upregulation in comparison to T/T mice and *GABRB3*, which was slightly increased in the hippocampus of KO mice (Fig. 5c, d).

## Discussion

In this study, we unexpectedly discovered a severe decrease in the size of hippocampal formation in mice as a result of SK3 potassium channel overexpression. In addition, in SK3-T/T mice there was a neuronal cytoarchitecture modification in the CA layers of hippocampus with a decrease and disorganization of neurons in CA1/CA3 hippocampal subfields. SK3-T/T mice also presented with LTP deficits in the hippocampus and failed to show novel object preference, which corroborates indications of an impairment of recognition memory, a subcategory of declarative memory. Declarative memory impairment is a known behavioral consequence of hippocampal damage [39].

Bond et al. [18] did not report the hippocampal anomaly although their doxycycline-induced conditional SK3-deficient adult mice were kept without doxycycline for at least 5 days in order to overexpress the SK3 channel. In our study, the conditional overexpressing SK3 mouse lineage did not receive doxycycline at all during embryogenesis, through development to adulthood. Therefore, the discontinuation or no administration of doxycycline in different time periods and at different stages of development may explain the differences between studies. Otherwise, it seems surprising that mice partly lacking the hippocampus survive and also do not suffer from dramatic memory deficiency. This phenomenon was also observed in humans whose hippocampi were almost entirely removed by surgery in early attempts to cure severe epilepsy. Such patients survived and mainly showed selective memory deficits [40].

The described features corroborate that SK3-T/T mice exhibit a profile of memory impairments. Previous results have also shown that increased SK3 channel expression in the hippocampus of old mice contributes to the age-dependent decline in learning, memory, and synaptic plasticity [9]. SK channel activation by the compound CyPPA (cyclohexyl-[2-(3,5-dimethyl-pyrazol-1-yl)-6-methyl-pyrimidin-4-yl]-amine, a positive modulator of the small conductance Ca^2+^-activated K^+^ channels SK2 and SK3) impairs learning and LTP [41]. Conversely, blockage of SK channels with apamin during a 5-Hz burst of tetanus facilitated the induction of LTP in the CA1 area [42]. Apamin facilitates long-term potentiation and encoding of memory traces [6, 43, 44].

Unpublished results from our laboratory suggest that the almost complete loss of hippocampal structures in adult SK3-T/T mice may be due to an early developmental defect before birth. SK3-T/T mice have been shown to exhibit an intrauterine growth-restricted phenotype [45]. As gestation progressed in T/T mice, litter sizes were reduced by more than one half by gestational days 13–14 and often exhibited fetal demise. This suggests that SK3 expression affects fetal development [45]. SK3 channel shows major expression changes during the perinatal and postnatal period compared with the adult brain regions [4, 46]. High levels of SK3 mRNA were observed in the subventricular zone up to embryonic days E19–E21 and in the intermediate zone up to E17 [46]. Bayer and Altman [47] have shown that the major hippocampal neuronal populations of the CA1 and CA3 subfields are generated between E15 and E20, when SK3 expression is high. It shows an evident parallelism of SK3 expression and cell organization in the hippocampal formation during embryogenesis.

Bates [48] describes by what mechanism could ion channel function contributes to the proper and timely migration of cells. McFerrin and Sontheimer [49] suggest that different currents (localized K^+^ and Cl^−^) could be important for cellular migration by helping the cell change shape as its volume increases at the leading edge and contracts at the trailing edge. Similarly, the disruption of the chloride *ClC*-*3* channel in mice resulted in a progressive degeneration of hippocampal formation [50, 51] that starts around postnatal day P12 and led to near complete loss of the hippocampus in the adult *Clcn*-*3* knockout mice [50]. Alternatively, SK3 excess could induce structural changes in hippocampal neurons during late phase of neuronal differentiation. SK3 channels are located in both the pre- and postsynaptic compartments of hippocampal pyramidal neurons [52], as a complex with Abelson interacting protein 1 (Abi-1) and the neural Wiskott Aldrich Syndrome Protein (nWASP) [53]. Furthermore, SK channels could have a protective role by counteracting calcium mobilization, central to excitotoxic injury. Persistent activation of SK channels might nonetheless tonically hyperpolarize neurons and reduce their spontaneous activity to induce the observed hippocampus deformation. In summary, it is tempting therefore to speculate that SK3 channel overexpression may regulate, directly or indirectly, the hippocampal formation development and function.

During our studies on the impact of SK3 overexpression on major brain neurotransmitter pathways, we observed a marked increase of DA receptors DRD1A and DRD2 in the frontal cortex and dorsal striatum and of DRD2 in the mesencephalon. SK3 modulates spike frequency in DA neurons [2, 5, 54]. Dopaminergic neuronal dysfunction is a key early event in Parkinson’s disease [2, 55] and in Huntington’s disease [56] progression, and perturbations in DA signaling are also implicated in the pathologies of attention-deficit hyperactivity disorder and schizophrenia.

Besides DA receptors, components of the DAergic/5HTergic and GABAergic/GLUergic pathways whose expression is increased in the hippocampus of SK3-T/T mice (see Table 1) have been linked to schizophrenia, Alzheimer’s disease, epilepsy, or autism. Interestingly, we observed a major increase of *GABRB3* mRNA in the hippocampus of T/T mice. Mutations in subunits of GABA receptors have been frequently associated with epilepsy, autism, and other neuropsychiatric disorders [75, 76]. In postmortem brains of schizophrenia patients, many changes in the GABAergic neural system have been reported, including the increase in GABA-A receptor expression [97], the decrease in GABA transporter expression [98], and the decrease in activity and mRNA content of glutamic acid decarboxylase [99]. The loss of GABAergic axons, which modulate hippocampal network activities, is a component of the core feature of disease-memory impairment [100]. SK3-T/T mice also showed a major increase of *APP* mRNA expression in the shrunken hippocampus. APP is a key protein associated with Alzheimer’s disease and is involved in the migration of neuronal precursor cells [101]. Patients with Alzheimer’s disease are characterized by a higher average rate of hippocampal volume loss than healthy age-matched controls [102, 103]. Experimental models of amnesia show that SK channel activity is implicated in memory impairment [104]. It is already known that damage arising from *APP* causes a subcellular redistribution of disrupted-in-schizophrenia 1 protein (*DISC1*) in primary cortical neurons, which in turn cannot properly migrate into the cortical plate [93]. Therefore, we speculate that the observed increase of *APP* mRNA in the SK3 overexpressing mice could be a reason for the organizational disruption of hippocampal layers.

**Table 1:** Altered DA/5HT and GABA/GLU genes in mice overexpressing SK3 channels and selected references describing their involvement in diseases

PCR array detected gene change	Described function	Alzheimer’s disease	Schizophrenia	Other diseases
AKT3 Thymoma viral proto-oncogene 3	Activation of the AKT system is specifically associated with hippocampal volume in first-episode schizophrenia.	No items found	[57]	[58, 59]
GSK3A Glycogen synthase kinase 3 alpha	Regulates production of Alzheimer’s disease amyloid-beta peptides.	[60–62]	[63]	[64, 65]
HTR2A 5-Hydroxytryptamine (serotonin) receptor 2A	Belongs to the serotonin receptor family; G protein-coupled receptor; mediates the action of antipsychotic drugs.	[66, 67]	[68, 69]	[70]
GABRB3 Gamma-aminobutyric acid (GABA)-A receptor, subunit beta 3	Is one of the subunits of a multi-subunit chloride channel that serves as the receptor for GABA; a candidate gene for autism.	[71, 72]	[73, 74]	[75–78]
GRIA2 Ionotropic glutamate receptor AMPA2	Functions as ligand-activated cation channel.	[79]	[80]	[81–83]
SLC1A2 Excitatory amino-acid transporter 2 or solute carrier family 1 member 2	Clears the excitatory neurotransmitter glutamate from the extracellular space at synapses.	[84, 85]	[86–88]	[89, 90]
APP Alzheimer’s disease amyloid beta (A4) precursor protein	Is the main component of the amyloid plaques found in the brains of patients with Alzheimer’s disease.	[91, 92]	[93, 94]	[95, 96]

In a more recent analysis of a schizophrenic samples, we found an association between the long CAG repeats (which reduce the SK3 potassium channel’s functioning) and better cognitive performance in tasks that assessed the ability to discriminate, select, and execute [10]. In addition, similar to SK3-T/T mice, brain analysis from schizophrenia patients have revealed hippocampal atrophy, neuron loss [105], reduced neural size [106], structural and histopathological alterations such as dendritic changes in the pyramidal neurons [107], and alteration of specific subtypes of interneurons [108].

In conclusion, the findings of the present study lead us to the hypothesis that the functional state of SK3 ion channels is a factor in determining directly or indirectly, morphological, molecular, and electrophysiological changes in the brain. Based on the mRNA expansion profiles we observed, we predict that transgenic mice that overexpress the murine SK3 gene may represent a research model for neuropsychiatric disorders, even though the symptoms of most the diseases cannot be exactly mirrored in mice. This potentially investigative model is of particular interest because progress in the understanding of the role of the SK3 channel in brain disorders has been limited due to the lack of specific SK3 antagonists and agonists. Our data also demonstrates that pharmacological modulation of SK3 could conceivably be beneficial for a range of central nervous system disorders and, as such, suggests that SK3 could represent a potential drug target.

## Electronic Supplementary Material

Below is the link to the electronic supplementary material.

Supplementary Movies 1a and 1b Movie of microCT scans from rostral to caudal parts of the brain. Video of coronal sections from microCT Z-stack scans in the brain of WT (**a**) and T/T mouse (**b**). Note the morphological differences of T/T hippocampus in comparison to WT (MPG 1764 kb)

(MPG 1772 kb)
